# Testing for soil-transmitted helminth transmission elimination: Analysing the impact of the sensitivity of different diagnostic tools

**DOI:** 10.1371/journal.pntd.0006114

**Published:** 2018-01-18

**Authors:** Marleen Werkman, James E. Wright, James E. Truscott, Alice V. Easton, Rita G. Oliveira, Jaspreet Toor, Alison Ower, Kristjana H. Ásbjörnsdóttir, Arianna R. Means, Sam H. Farrell, Judd L. Walson, Roy M. Anderson

**Affiliations:** 1 London Centre for Neglected Tropical Disease Research (LCNTDR), Department of Infectious Disease Epidemiology, St. Mary’s Campus, Imperial College London, London, United Kingdom; 2 The DeWorm3 Project, The Natural History Museum of London, London, United Kingdom; 3 Helminth Immunology Section, Laboratory of Parasitic Diseases, National Institute of Allergy and Infectious Disease, National Institutes of Health, Bethesda MD, United States of America; 4 Department of Global Health, University of Washington, Seattle, Washington, United States of America; Evidence Action, AUSTRALIA

## Abstract

In recent years, an increased focus has been placed upon the possibility of the elimination of soil-transmitted helminth (STH) transmission using various interventions including mass drug administration. The primary diagnostic tool recommended by the WHO is the detection of STH eggs in stool using the Kato-Katz (KK) method. However, detecting infected individuals using this method becomes increasingly difficult as the intensity of infection decreases. Newer techniques, such as qPCR, have been shown to have greater sensitivity than KK, especially at low prevalence. However, the impact of using qPCR on elimination thresholds is yet to be investigated. In this paper, we aim to quantify how the sensitivity of these two diagnostic tools affects the optimal prevalence threshold at which to declare the interruption of transmission with a defined level of confidence. A stochastic, individual-based STH transmission model was used in this study to simulate the transmission dynamics of *Ascaris* and hookworm. Data from a Kenyan deworming study were used to parameterize the diagnostic model which was based on egg detection probabilities. The positive and negative predictive values (PPV and NPV) were calculated to assess the quality of any given threshold, with the optimal threshold value taken to be that at which both were maximised. The threshold prevalence of infection values for declaring elimination of *Ascaris* transmission were 6% and 12% for KK and qPCR respectively. For hookworm, these threshold values are lower at 0.5% and 2% respectively. Diagnostic tests with greater sensitivity are becoming increasingly important as we approach the elimination of STH transmission in some regions of the world. For declaring the elimination of transmission, using qPCR to diagnose STH infection results in the definition of a higher prevalence, than when KK is used.

## Author summary

Soil-transmitted helminths are categorised as a neglected tropical disease and comprise four dominant species (two hookworms, *Trichuris trichuria* & *Ascaris lumbricoides*) that affect the poorest people in the world. The World Health Organisation (WHO) has made great strides in reducing the morbidity induced by STH infections in pre-school aged and school-aged children through mass drug administration. Many countries are now considering moving from morbidity reduction in school-aged children to community-wide treatment with the aim of transmission elimination. These helminths reproduce sexually within a human host and therefore both male and female worms must be present to produce fertilized eggs. The density of female and male worms below which mating success is too low to sustain parasite populations is defined as the ‘breakpoint’ in transmission. Both the prevalence and intensity of infection are very low as this breakpoint is approached when worm numbers are highly aggregated in their distribution within the human host population. Consequently, it becomes increasingly challenging to identify infected individuals using standard microscopic diagnostic tools (such as Kato-Katz). New and more sensitive molecular diagnostics tools, such as qPCR, are a necessity in settings where communities are moving towards the interruption of transmission. This paper demonstrates that the threshold to declare interruption of transmission is 50% lower when microscopic techniques are applied compared with molecular techniques.

## Introduction

Since the launch of the London Declaration of Neglected Tropical Diseases in 2012, much progress has been made to reduce both the prevalence and associated morbidity of the neglected tropical diseases (NTDs), including lymphatic filariasis (LF), schistosomiasis, onchocerciasis and the soil-transmitted helminths (STHs) [1]. STHs are the most prevalent NTD globally and are often co-endemic with other helminth infections. STH species include hookworm (*Necator americanus*, *Ancylostoma duodenale* and *Ancylostoma ceylanicum*), roundworm (*Ascaris lumbricoides*) and whipworm (*Trichuris trichiura*). These species remain endemic in large parts of the world, mainly affecting the poorest regions of sub-Saharan Africa and South-East Asia [2,3]. Although infection with STH rarely results in death, high worm burdens in children are thought to affect growth, impair intellectual development and cause anaemia, resulting in an estimated 5 million disability-adjusted life years lost (DALYs) [2,4].

Currently, the World Health Organisation (WHO) aims to treat 75% of pre-school-aged children (pre-SAC: 2–4 years) and school-aged children (SAC: 5–14 years) in high-risk areas by 2020 [5]. However, this target has not yet been achieved in many areas [3]. With morbidity occurring more frequently amongst children than adults (excepting hookworm infection in pregnant women), the latter are normally excluded from mass drug administration (MDA) programmes. Moreover, SAC are easy to reach when they attend school and, with limited resources for public health available in many STH endemic regions, it is a cost-effective method to reach a large proportion of the population. However, if adults remain untreated, they will continue to contribute to the reservoir of infectious eggs or larvae, resulting in children being continually re-infected form this infectious reservoir[6–8]. Targeted treatment of SAC-only is, therefore, unlikely to result in transmission interruption. Continued treatment with MDA will remain a necessity to control the worm burden amongst SAC unless major improvements to water, sanitation and hygiene (WASH) infrastructure occur through economic growth that benefits the rural poor as well as the middle classes in urban areas [9,10]. Anthelmintic drugs are currently donated by the major pharmaceutical companies (GlaxoSmithKline and Johnson & Johnson) as promised in the London Declaration. However, the continuation of large scale drug donations beyond 2020 remains uncertain [1]. Therefore, initiatives that explore the feasibility of interrupting transmission are important at present [11].

Two ongoing studies investigating the feasibility of interruption of STH transmission by MDA only are the TUMIKIA project [12] and the DeWorm3 project [13]. The countries that are involved in these projects (Kenya for TUMIKIA; Benin, Malawi, and India for DeWorm3) have run successful MDA elimination programmes for LF and therefore have a strong foundation upon which to build an STH elimination programme. LF is treated by albendazole or mebendazole in combination with either ivermectin or diethylcarbamazine. Both albendazole and mebendazole are also effective in reducing the prevalence and infection intensity of STH [13,14]. Moreover, MDA programmes for the elimination of LF are provided to the whole community and therefore offer a unique opportunity to upscale STH MDA programmes from targeting pre-SAC and SAC to community-wide initiatives. The DeWorm3 project was specifically designed to leverage the successes of the LF programmes to increase the likelihood of STH elimination.

STH parasites are dioecious and reproduce sexually within the human host. Consequently, both sexes need to be present within a single host to produce fertilized eggs. Hosts subsequently expel the (fertilized) eggs into the environment, where (re-)infection of other hosts can occur [15]. As both STH prevalence and worm burden decrease, the likelihood of both sexes residing within a single human host declines. Therefore, critical prevalence and average intensity thresholds exist at which the number of human hosts with both male and female worms falls below a critical fraction of the population, resulting in a breakpoint of transmission [15]. When this breakpoint is crossed, the parasites cannot reproduce frequently enough to sustain transmission and the population of worms will decay to extinction, even in the absence of further treatment.

Various studies have investigated the optimal prevalence threshold, under defined MDA regimes, at which to reliably detect (with a defined level of confidence) the interruption of transmission for hookworm, and the time post-cessation of treatment at which prevalence should be measured [9]. Truscott et al. suggested that 1–2% prevalence, as determined by two independent stool samples analysed through the McMaster technique and measured one to two years after the last round of MDA, is a good indicator of STH transmission interruption [9]. However, detecting infected individuals using standard diagnostic tools is challenging at low prevalence levels. Low worm burdens and limited egg output complicate the detection of STH species, as the number of eggs in the stool reduces when the number of worms in the host decreases [16]. Studies show, through repeated sampling of a single stool, that many false negatives are recorded at low prevalence levels [17,18].

Multiple diagnostic tools are available to test for the presence of STH. Kato-Katz (KK) is one of the most commonly applied methods, as it is relatively cheap and easy to apply in resource-limited settings [19]. Moreover, KK is also applied to diagnose other helminth infections such as the schistosomes [20]. KK is a measure of the egg output in stool and a moderate-to-high correlation exists between egg output and worm burden [16]. However, due to the density dependence of worm fecundity, egg count is a poor proxy measure for worm burden. Previous studies have shown great heterogeneity in epg counts across multiple stool samples due to the aggregated distribution of eggs with the stool, stool composition and day-to-day variability within a given host [18]. Further, as worm burden reduces with prevalence, so too does the sensitivity of KK [21]. Multiple slides and/or stool samples collected over multiple days can increase the sensitivity of KK [22], as the egg output per host is highly variable [23]. However, the availability of skilled technicians plus economic considerations often limits the number of slides that can be examined, especially in the design of Monitoring and Evaluation (M&E) programmes in large national programmes or trials.

New techniques (for helminthology), such as the quantitative polymerase chain reaction (qPCR), are a promising alternative to KK. Such techniques have been in use for other infectious agents for some time but have been slow to be introduced in helminthology. With qPCR, the DNA of helminth eggs in faeces is amplified and, unlike in standard PCR, this can be translated to a quantitative measure of the infection intensity. qPCR has been found to be more sensitive than KK, particularly in low prevalence settings [16]. This is of particular importance in the context of elimination, due to the low prevalence and reduced worm burdens expected as the transmission breakpoint is approached. Due to the expected low worm burdens in these settings, KK would have lower sensitivity whilst qPCR is more likely to detect the very lowest of intensities. Recently, qPCR has also been shown to exhibit less measurement error (less variation between readings) compared with KK [17]. Molecular techniques have also been shown to have a higher specificity as they are able to distinguish between hookworm species [24,25].

KK remains the standard diagnostic tool implemented in STH morbidity control programmes due to its continued presence in WHO guidelines despite its low sensitivity. This is in part due to the low cost of the technique. Nevertheless, with the shift in focus from morbidity control in SAC to elimination through interruption of transmission in entire communities, there is a need for more sensitive diagnostic tools. At present, however, the impact of qPCR on defining the elimination of transmission has yet to be investigated. In this paper, we aim to quantify the impact of using qPCR on measured prevalence as opposed to KK, in a population approaching elimination. We employ epidemiological data and mathematical models of transmission and MDA impact in our analyses. We focus these analyses on the impact of the sensitivity of different diagnostic tools on measuring the optimal prevalence threshold at which to declare the interruption of transmission.

## Methods

### Data

In this paper, data are utilised from an STH epidemiology study performed in five villages in western Kenya [16]. The objective of this study was to measure the sensitivity of modern molecular tools (qPCR) to examine the intensity of infection and prevalence of STH (all three of the major infections) and to compare this with standard KK analysis [16]. All inhabitants of the five villages were approached to participate in this study, though the number of households targeted for stool sample collection was reduced, and those included were selected at random. At the start of the study, stool samples were collected from 1551 enrolled individuals and were examined with KK (duplicate smears on two different days). All the participants with positive KK results were treated with 400 mg of albendazole and their stool was collected from days 2 to 7 post-treatment. Worms (*Ascaris*) were collected from the stool and the number of worms per individual was recorded. It is estimated that approximately 80% of worms were expelled from each individual in this period, based on a pilot study done by the research group and previous work by others [16,26,27]. All positive individuals plus an additional 10% of participants who were not found to be positive, and represented the control group, were included in the first *Ascaris* expulsion study (n = 4773). All remaining, willing members of the five study villages (n = 3687) were then treated with 400 mg of albendazole. Seventy-four participants with a positive *A*. *lumbricoides* egg count by KK provided stool samples 3 weeks post-treatment to test treatment efficacy. All participants had negative KK results at this time point, suggesting that treatment was successful. A follow-up study was performed three months post-treatment during which stools were collected from 1227 individuals, and worm expulsion was performed on 66 individuals. Samples from both the baseline and follow-up study were examined with KK and qPCR (n = 1884 samples from 796 people with measurements by both KK and qPCR).

### Model description

We implemented a stochastic, individual-based model of parasite transmission and treatment, details of which can be found in past publications [28–30]. The model simulates the worm burden of individual hosts within a community (i.e. village) undergoing MDA and its structure is founded on a deterministic model based on a set of partial differential equations [6,8,30]. The model parameter values that were adopted in this study are shown in S1 Table. S1 Fig compares the stochastic individual-based model with the deterministic model as described in Truscott et al., 2014 and 2016 [6,8,30]. With non-linear systems, the mean of a stochastic formulation is not necessarily equal to the deterministic prediction. But the non-linearity in this helminth model formulation are not so severe that this pattern arises. As can be seen in S1 Fig, the mean of the stochastic model converges to the deterministic result as the number of simulations rises. Hosts become infected when exposed to the infectious reservoir of eggs in the habitat, to which infected hosts contribute both fertilized and unfertilized eggs. These contributions are age-dependent and the precise pattern of this dependency varies between the STH species [6,31]. In the case of *Ascaris*, children dominate the contribution to the infectious reservoir whilst, in contrast, the highest hookworm burdens are found amongst adults [32]. The age profile of infection of *Ascaris* is fitted to data collected during a study performed in the 1980s in southern India [30,33], while the hookworm data are fitted to epidemiological measurements collected in Vellore, India in 2013–15 [34,35]. The worm burdens in hosts (and egg output) follow a negative binomial probability distribution, which is typically observed for all helminth species in humans and other mammalian hosts [18,30,36–38]. This assumption means that a large proportion of people will have few worms, and a small proportion of people suffer from very high worm burdens. The negative binomial parameter *k*, which varies inversely with the degree of worm aggregation, is used to describe the degree of clumping. The relationship (from the negative binomial probability generating function) between the proportion of infected individuals (*P*) and mean worm burden (*M*) is given by the following equation:

$$P=1-{\left(1+\frac{M}{k}\right)}^{-k}$$

In this study, we set *k* as 0.285 (S2 Fig) and 0.35 [35] for *Ascaris* and hookworm, respectively. For Ascaris, data from Easton et al. [16] was applied to derive estimates of the aggregation parameter *k*. We included records from individuals who participated with the worm expulsion study (n = 205). However, individuals with an egg count>0 but no expelled worms were excluded from this analysis (n = 38) to fit a negative binomial distribution (S1 R Code).

Due to the breakpoint phenomenon, it is not necessary to eliminate all worms. Breakpoints for transmission as measured by the mean worm burden have been estimated for LF, onchocerciasis and, more recently, for STH and schistosomiasis [9,39,40]. The performance of prevalence thresholds can be quantified through stochastic simulation models or in real field settings within studies. The positive predictive value (PPV) and negative predictive value (NPV) have been shown to be good measures for quantifying the quality of a given threshold in previous studies [9,10]. The PPV is the proportion of simulations (or villages or clusters in a field trial) achieving elimination, and that are identified as achieving elimination, divided by all the simulations (villages or clusters) that do achieve elimination based on the prevalence achieved two years post-MDA. The NPV is defined as the proportion of simulations that bounce back to the endemic levels recorded or defined prior to treatment, that are identified as bounce-backs, divided by the simulations that are identified as bounce-backs. For the purpose of this study, we assume that these communities behave as independent units, consequently migration was not included in this study. Migration patterns are thought to be important as individuals with STH infections can contaminate the environmental reservoir with open defecation. A modelling study showed that the likelihood of elimination reduces substantially when the number of immigrants increases [10]. In the context of the endgame, molecular data could be useful in investigating who is infecting whom (i.e. form with a village population of from another village). However, real data describing migration patterns in the context of STH transmission is currently lacking. It is certainly likely to prolong transmission in areas, particularly if there is migration of infected individuals (such as migrant labourers) who are not treated. A practical solution would be to recommend migrants to receive treatment of albendazole upon arrival in their home village [10].

We ran 1000 simulations, and for each simulation we construct a village of 1000 people. Age-dependent births and deaths of the human hosts are included, and follow the demography of a typical low-income country [41]. The simulations are run for 50 years and include 10 endemic years (assuming no treatment), four years of LF MDA community-wide treatment and three years of STH community-wide treatment. We varied the coverage and frequency of the treatments for *Ascaris* and hookworm. To establish the optimal prevalence threshold to declare elimination as reflected as the point where the PPV and NPV intersect, we must ensure that not all simulations result in elimination. Each species requires a different MDA coverage and frequency of treatment programme to get near to the breakpoint. To a large extent, this depends on parasite life expectancy (easier for longer lived species) and the magnitude of the basic reproductive number R_0_ (see S1 Table for an overview of parameter values selected for this study). For *Ascaris*, we applied annual treatment with a coverage of 70% in pre-SAC and SAC and 35% in adults. For hookworm, we modelled biannual treatment with a coverage of 70% for pre-SAC and SAC, and 60% for adults. Table 1 shows an overview of the study including timings and frequency of treatment. Individuals are chosen at random at each time point of MDA delivery (we assume there is no systematic non-adherence) stratified by population size in each age group. We assume that no treatment is provided after the last round of MDA for STH. At low prevalences, the dynamics of transmission are dominated by chance events (stochastic processes) and it often takes many years for some populations to bounce back or finally eliminate their parasites. Therefore, we run each simulation for 50 years to allow for sufficient time to observe any bounce-backs. At year 50, we examine if the prevalence has bounced back to endemic levels or if transmission was truly interrupted.

**Table 1:** Overview of the treatment programme included in the simulation study.

Phase	Description	Duration	Treatment
I	Endemic period	10 years	No mass drug administration (MDA) treatment
II	Lymphatic filariasis treatment programme	4 years	Annual community-wide MDA with a coverage of 70% in pre-SAC, 70% in SAC and 60% in adults.
III	Intensified soil-transmitted helminth treatment programme	3 years	*Ascaris*: annual MDA treatment with a coverage of 70% in pre-SAC, 70% in SAC and 35% in adults. Hookworm: biannual MDA treatment with a coverage of 70% in pre-SAC, 70% in SAC and 60% in adults.
IV	Recovery period	2 years	No MDA treatment
V	Interruption or bounce back	At year 50	No MDA treatment

**Fig 1:**
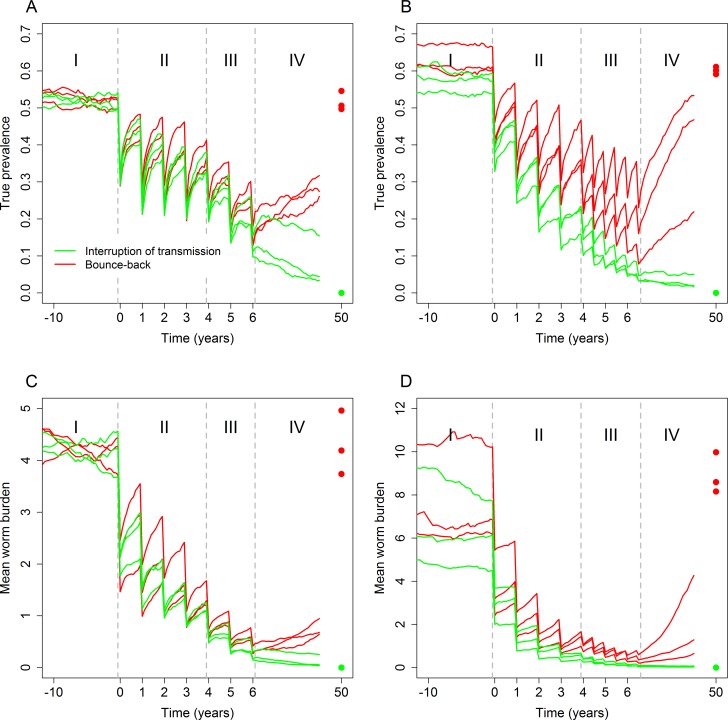
Simulated prevalence for *Ascaris* (A) and hookworm (B) over time and mean worm burden for *Ascaris* (C) and hookworm (D) during different phases of a study for six model simulation runs (three achieving interruption of transmission and three simulations that bounce-back to endemic levels). Phase I is the endemic phase and consists of 10 years, Phase II is the LF treatment (MDA) phase with annual community-wide treatment for four years, Phase III is the intensified STH treatment for three years and in Phase IV no treatment is provided to the community. The dots represent the prevalence at year 50.

Fig 1 illustrates the effects of the STH MDA treatment (as defined in the simulations) on the true prevalence of *Ascaris* (Fig 1A) and hookworm (Fig 1B). In this paper, true prevalence is defined as the proportion of individuals with at least one worm (either male or female). We present the time series of the STH elimination programme, including the endemic period (10 years), LF community-wide treatment, and intensified community-wide STH treatment followed by a period of no treatment. The results plotted in Fig 1 illustrate three simulations that achieved interruption of transmission and three simulations that did not achieve this goal, subsequently bouncing back to endemic prevalence levels.

### Diagnostic model

The diagnostic tools examined in this study are based on the detection of helminth eggs in a faecal sample from patients. The production of fertilized eggs is only possible when a male and female worm are within the same host. However, female *Ascaris* worms produce (unfertilized) eggs even in the absence of male worms [30,42,43]. In contrast, female hookworms are thought to only release eggs fertilized after mating [30]. These factors clearly affect measured prevalence at low worm burden intensities, as the likelihood of having both male and female worms in the same host decreases. To investigate the prevalence in a population, 250 random individuals from the whole community were selected. In this study, we are only interested in the absence or presence of worms within a human host, and not the intensity of infections in individuals. Therefore, we adopt a simple approach for detection, and use an egg detection probability based on the data collected by Easton et al., [16]. A host has a probability of 0.98 to be found positive with qPCR if they have at least one female worm (*Asc*aris) or have both a male and female worm (hookworm). The data collected by Easton et al., [16,17] was also designed to investigate measurement error in the readings. Multiple readers/technicians were used to analyse different KK slides prepared using samples from the same hosts, and these samples were tested repeatedly by qPCR. As a result, there are up to 8 KK slides and up to 20 qPCR readings per host (i.e. per faecal sample) per time point (baseline and follow-up). To test for KK positivity, an individual found positive with qPCR was selected at random. For that chosen individual, two of their KK results were selected at random (without replacement). If at least one these samples had one or more eggs, the individual was identified as positive.

Based on previous studies, we assume that the specificity is 100% for both KK and qPCR (i.e. there are no false positives) [24,44,45]. As the sensitivity of the diagnostic tools differ both across species and between studies, an analysis was performed to investigate whether this has an impact on the value of the prevalence threshold to declare transmission elimination as suggested by high PPV and NPV values.

## Results

To investigate the best prevalence threshold for declaring that the parasite population has crossed the breakpoint in transmission, and hence transmission has been interrupted, it was important to ensure that not all simulations achieve elimination. In the case of *Ascaris* 64% of the simulations achieved elimination whilst for hookworm this value was 47%. In this study, the negative binomial aggregation parameter, *k*, was set to 0.285 for *Ascaris* and 0.35 for hookworm. The transmission parameter, R_0_, was fixed at 2.12 for *Ascaris* and 2.2 for hookworm. Fig 1 shows how the prevalence changes during the four phases of the programme. The mean (true) prevalence during the endemic phase is approximately 52% for *Ascaris* (Fig 1A) and 60% for hookworm (Fig 1B). If interruption of transmission is not achieved, the infection levels bounce-back to endemic levels (Fig 1C and 1D).

**Fig 2:**
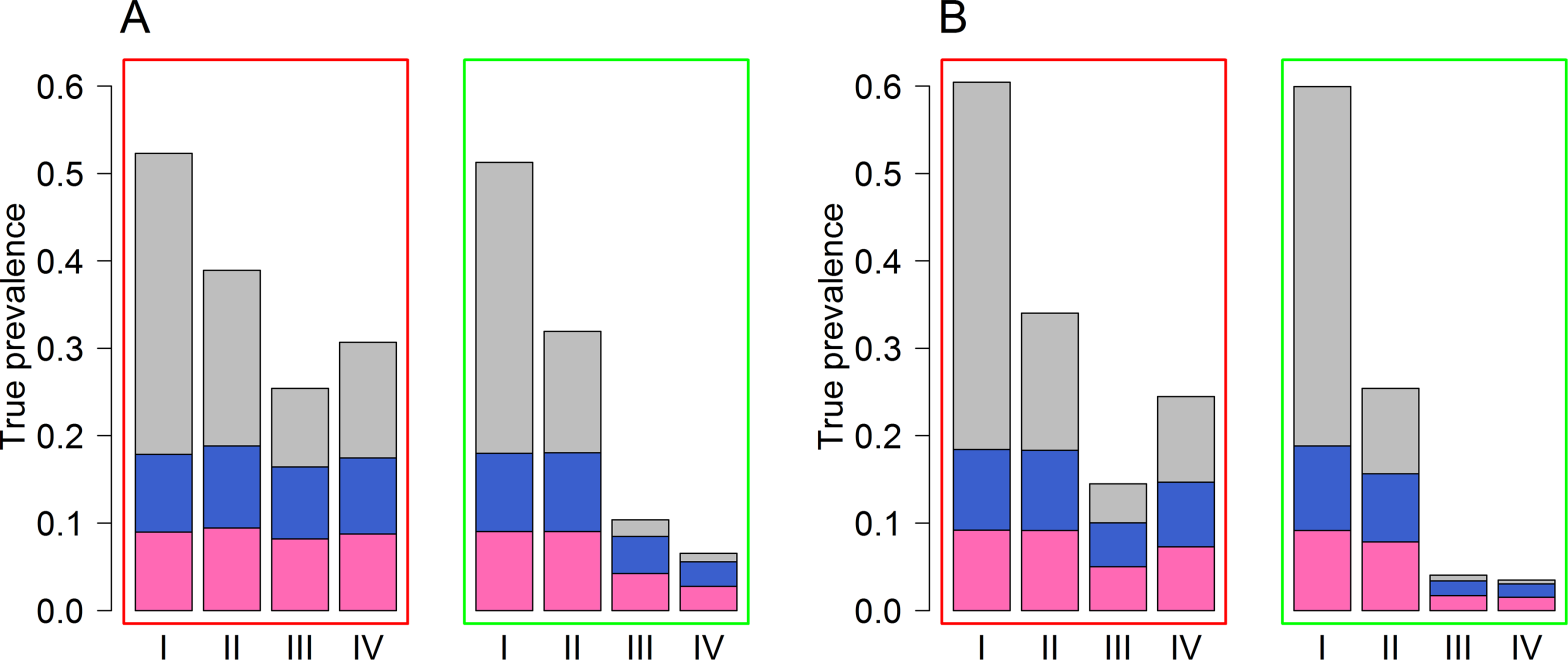
Average proportion of hosts who have only female worms (pink), only male worms (blue) and hosts who have both sexes (grey). Fig 2A shows results for *Ascaris* and Fig 2B shows results for hookworm. Results within the red rectangles represent simulations that bounce back and results within the green rectangles represent the simulations that achieve interruption of transmission. The results are shown for four key time points: I: endemic state; II: LF Phase; III: STH Phase; IV: two years after last round of MDA (Table 1).

To achieve interruption of transmission, the number of individuals harbouring worms of both sexes needs to become so low that the likelihood of successful mating falls below the level required to maintain the parasite population. Fig 2 shows the combination of individuals with only male worms, only female worms, or worms of both sexes at four key time points, from our simulations of worm loads in individuals. These time points include the endemic state, post-LF treatment, post-STH treatment and two years after the last round of MDA (Table 1). In the simulations that bounce back to endemic levels, both the proportion and absolute number of hosts in which both sexes reside are higher in value compared with simulations that do achieve interruption of transmission (Fig 2). For example, in the *Ascaris* simulations which do not achieve interruption of transmission, the proportion of hosts in which both sexes reside reduces from 34% in the endemic phase to 20% during the LF phase and 9% in the STH phase, but then increases to 13% two years after the last round of MDA is provided (Fig 2A). However, for the *Ascaris* simulations that do achieve interruption of transmission, the proportion of hosts in which both sexes reside is 33%, 14% and 2% for the endemic phase, LF phase and STH phase. As prevalence also decreases when a village moves towards parasite elimination, the proportion of hosts that harbour both sexes, and thus can produce fertilized eggs, is reduced to <1% (Fig 2A). Similar results were obtained from the hookworm simulations (Fig 2B). If interruption of transmission is achieved for hookworm, then on average 0.4% of the hosts in the population are predicted to excrete fertile helminth eggs into the environmental reservoir from the simulations (Fig 2B).

**Fig 3:**
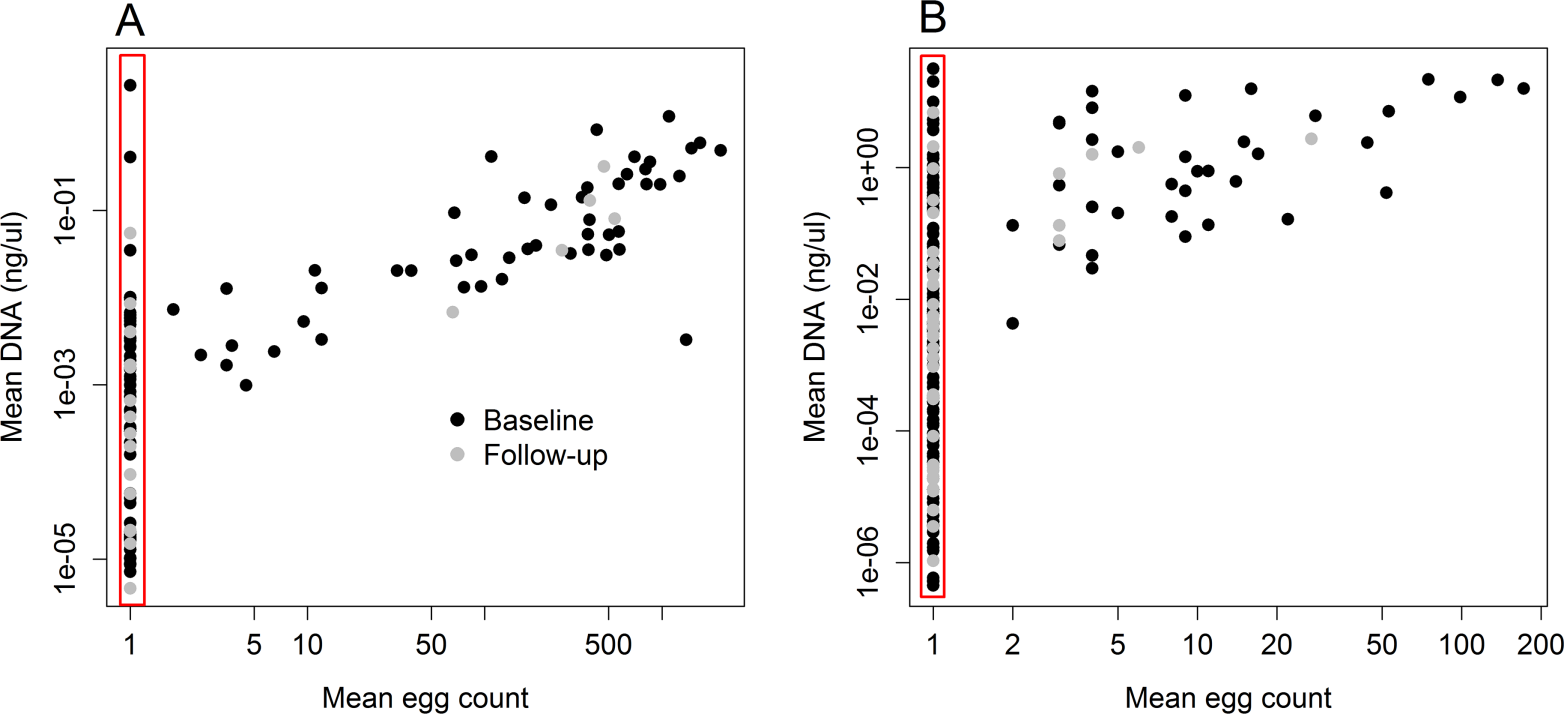
Correlation of the mean qPCR results and mean egg count (+1) results for *Ascaris* (A) and hookworm (B) data from Easton et al. 2016 [16]. The red square highlights the individuals with a negative KK result and positive qPCR result.

Fig 3. shows the relationship between mean DNA concentration (ng/μL) and the mean egg count + 1 for *Ascaris* (A) and hookworm (B) from the results recorded in Easton et al. [16]. The red square highlights the hosts that had zero egg count but were found positive with qPCR. All available samples from each host and each timepoint were included in this analysis, and the means were calculated. For *Ascaris*, 54 individuals were found positive with both methods, 74 were found positive with qPCR and negative with KK, and 3 individuals were negative with qPCR and positive with KK. For hookworm, 42 individuals were found positive with both methods, 145 were found positive with qPCR and negative with KK, and 3 individuals were negative with qPCR and positive with KK. The egg counts from KK and the DNA quality provided by qPCR show a strong positive relationship, we apply these data in this study to investigate the importance of diagnostic sensitivity on declaring interruption of transmission.

**Fig 4:**
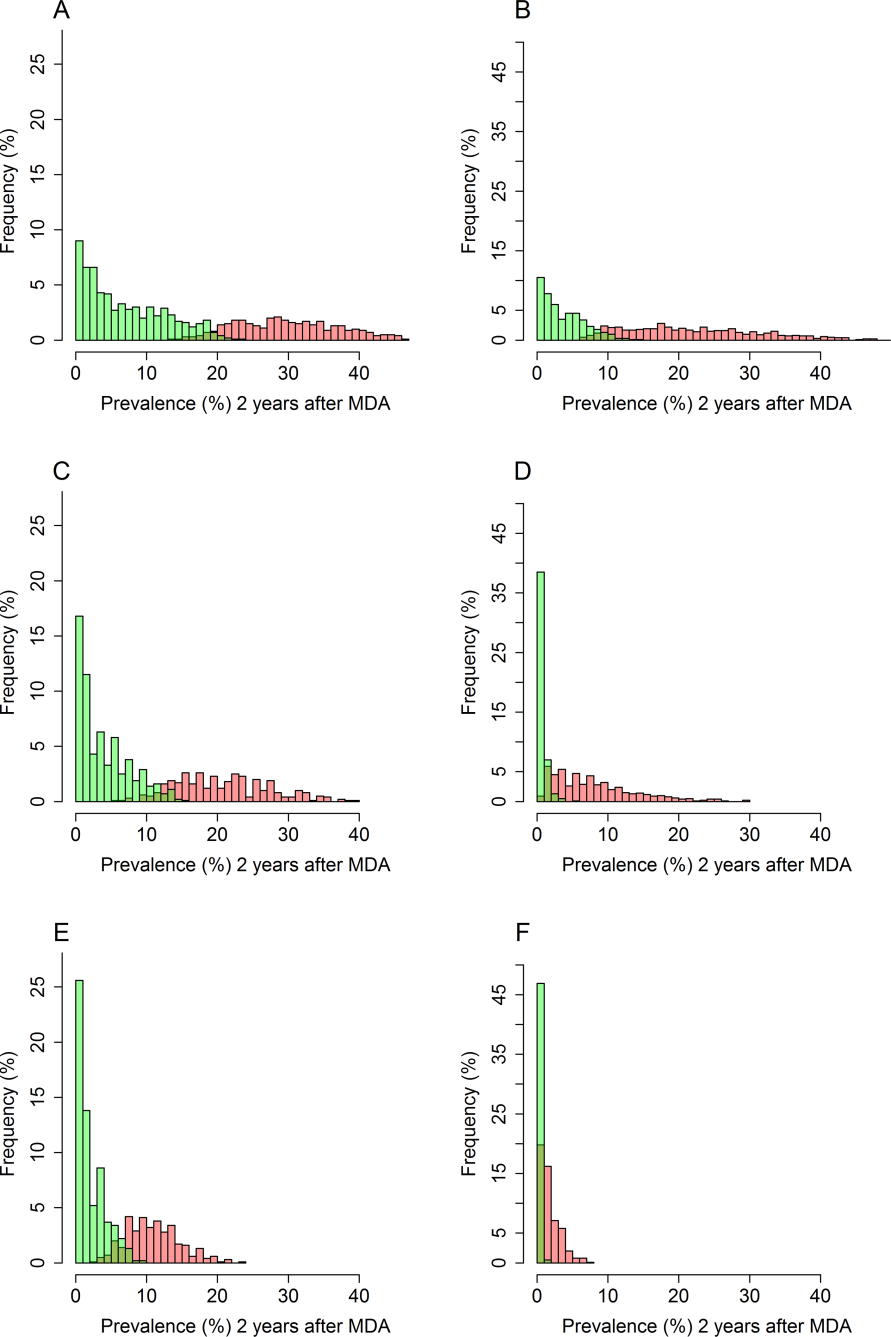
Histograms showing the true prevalence (A+B) from the simulations, the predicted measured prevalence with qPCR (C+D) and the predicted measured prevalence with KK (E+F), measured two years after the last round of treatment for *Ascaris* (A+C+E) and hookworm (B+D+F). Green bars represent the simulations that result in interruption of transmission and the red bars represent the simulations that result in bounce-backs.

Determining the threshold to declare interruption of transmission is not straightforward as there is no clear separation between the distribution of simulated endline prevalences for the entire community (2 years post-treatment) of simulations that achieve interruption of transmission and for simulations that bounce-back to the endemic state (Fig 1A and 1B). The PPV and NPV values can help to determine the optimal threshold since in an ideal world the values of both should be maximised. Fig 4 shows the distribution of prevalences measured two years post-MDA for *Ascaris* (Fig 4A, 4C and 4E) and hookworm (Fig 4B, 4D and 4F). If true prevalence (the proportion of individuals harbouring either one male worm, one female worm of worms of both sexes) could be measured, the optimal threshold for declaring elimination would be 20% for *Ascaris* and 9% for hookworm (Tables 2 and 3). However, as both KK and qPCR can only detect egg-producing female *Ascaris* and fertilized eggs for hookworm, the prevalence thresholds need to be substantially lower due to the lower sensitivity. Thus, the threshold value for interruption of transmission of Ascaris is 6% when KK is applied and 12% with qPCR is used (Table 2). For hookworm, these threshold values are lower, at 0.5% for KK and 2% for qPCR (Table 3). It is important to note that the PPV values are considerably lower for hookworm compared with *Ascaris*, especially for the KK method. This reflects the higher KK sensitivities for *Ascaris* found by Easton et al. [16], who found a KK sensitivity of 32% for hookworm and 70% for *Ascaris*. A higher threshold is beneficial as the sample size required to detect a threshold decreases as the threshold increases (S3 Fig)[46].

**Table 2:** Positive predictive value (PPV) and negative predictive values (NPV) of different thresholds for *Ascaris*. The highlighted green cells represent the optimal threshold for each method where the values for both measures intersect.

Threshold (%)	True Prevalence		Measured prevalence: qPCR		Measured prevalence: Kato Katz	
	PPV	NPV	PPV	NPV	PPV	NPV
3	1.00	0.46	1.00	0.54	1.00	0.67
4	1.00	0.48	1.00	0.59	1.00	0.78
5	1.00	0.52	1.00	0.63	0.97	0.83
6	1.00	0.54	1.00	0.69	0.96	0.92
7	1.00	0.57	1.00	0.73	0.91	0.94
11	1.00	0.68	0.98	0.91	0.77	1.00
12	1.00	0.71	0.97	0.95	0.75	1.00
13	1.00	0.76	0.94	0.96	0.72	1.00
17.5	0.98	0.89	0.83	1.00	0.65	1.00
20	0.96	0.97	0.79	1.00	0.65	1.00
25	0.86	1.00	0.70	1.00	0.64	1.00

**Table 3:** Positive predictive value (PPV) and negative predictive values (NPV) of different thresholds for hookworm. The highlighted green cells represent the optimal threshold for each method where the values for both measures intersect.

Threshold (%)	True Prevalence		Measured prevalence: qPCR		Measured prevalence: Kato Katz	
	PPV	NPV	PPV	NPV	PPV	NPV
0.5	1.00	0.56	0.98	0.79	0.77	0.93
1	1.00	0.58	0.97	0.85	0.71	0.99
2	1.00	0.64	0.91	0.96	0.59	1.00
3	1.00	0.69	0.80	0.98	0.52	1.00
5	1.00	0.78	0.72	1.00	0.48	1.00
8	0.97	0.91	0.61	1.00	0.47	*NA*
9	0.95	0.94	0.58	1.00	0.47	*NA*
10	0.90	0.96	0.57	1.00	0.47	*NA*

**Fig 5:**
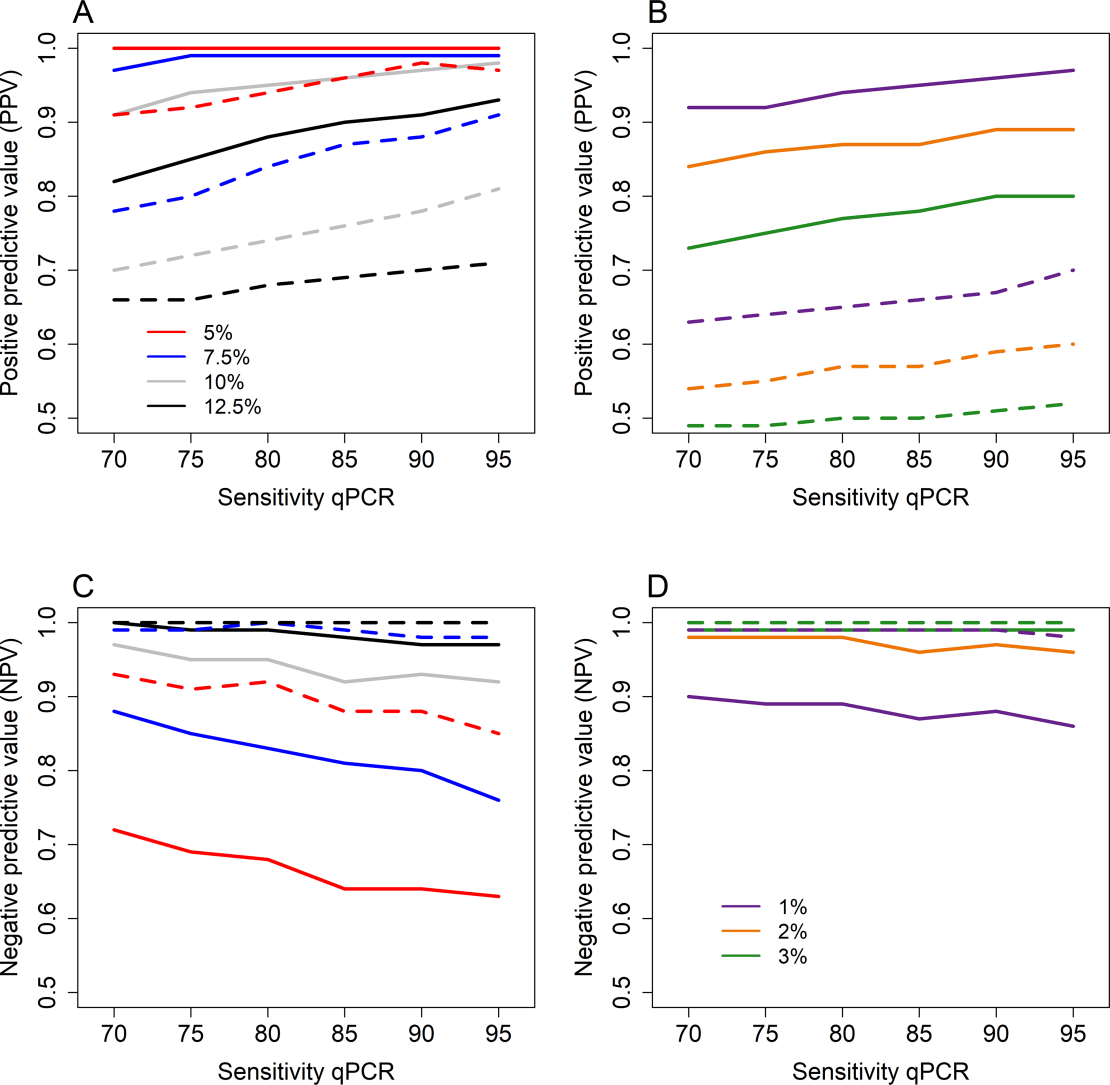
Results of the sensitivity analysis of the sensitivity of qPCR, showing the positive predictive values for *Ascaris* (A) and hookworm (B); and the negative predictive values for *Ascaris* (C) and hookworm (D). The solid lines show the PPV/NPV results of qPCR and the dashes lines the results of KK. Results are shown for differing prevalence threshold levels, 5–12.5% for Ascaris and 1–3% for hookworm.

Fig 5. shows the results of the sensitivity analysis, where the sensitivity levels of qPCR tools were varied from 70%-95%. The sensitivity of the KK results was kept relative to the qPCR result based on the data of Easton et al. [16] as described above. These results indicate that the PPV values for both species are more dependent on the prevalence threshold rather than the sensitivity of the diagnostic test (Fig 5A and 5B). For *Ascaris*, the PPV remains > 0.95 for qPCR sensitivity levels up to 70% if the prevalence thresholds < 10%. Whilst the PPV values for KK are > 0.8 for qPCR sensitivity levels > 75% (Fig 5A). The PPV values for hookworm remain > 0.85 if the prevalence threshold is 2% or lower and the qPCR sensitivity > 75%. The KK PPV values drop to 0.54 when the qPCR sensitivity is assumed to be 70% and the prevalence threshold is 2%. For hookworm, the NPV values are always > 0.95 if the prevalence threshold is 2% or higher for both qPCR and KK (Fig 5D). Whilst for *Ascaris* the NPV values are substantially lower for qPCR results compared with KK results (Fig 5C).

## Discussion

In this study, we have focused on the absence or presence of parasites in the human host and not the intensity of infection. However, the current WHO guidelines are based on morbidity control in SAC, and this is reflected within the guidelines by a series of classes for the intensity of infection, as measured by the number of eggs per gram (epg) of faeces, to reflect low, medium, and high intensities. Some published studies have suggested different methods to mimic the measuring of STH infection [30,47]. As mentioned previously, epg is not a direct measure of the number of female worms present in the host, as the number of eggs produced per female worm is known to exhibit strong density-dependence. Consequently, the total number of eggs produced per worm declines as worm burden rises [38,48,49]. This non-linearity can be accounted for within diagnostic models as illustrated by Coffeng et al. and Truscott et al. [30,47]. These models require worm expulsion data to estimate the two fecundity parameters, the density dependent parameter (γ), which measures the severity of the non-linearity, and the mean number of eggs produced per female worm in the absence of density dependent constraints (λ) [49]. Fitting these two model parameters requires acquiring worm expulsion data in combination with epg data from stools, such as KK, and to link epg output to the number of female and male worms within a host plus recording per capita egg output per female worm. When non-linear density dependent functions (power or exponential) are fitted to such data they show much variability due to errors in measurement in both epg and worms expelled [30].

Worm expulsion studies are highly demanding and time-consuming. Moreover, the results of the worm count are unreliable as it is difficult to collect all stools produced over a period of several days and, in the case of hookworm, find all the worms present in stool. To collect between 80% and 97% of the worms present within a host, stool needs to be collected from day 2 to at least day 7 post-MDA for all individuals [16,26,27]. However, compliance of stool donation varies between individuals and is negatively correlated with the length of the collection period. Therefore, the number of worms collected per individual is typically an underestimation of the true number of worms that are present in a host [27]. The individuals who were included in the worm study by Easton et al. (2016) had *Ascaris* worm counts ranging from zero up to 54 parasites per individual [16]. This is higher than the worm counts in the simulation data, therefore, it may be possible that the sensitivity reported in Easton et al. (2016) is overestimated at very low worm burdens.

However, our sensitivity analysis indicates that our findings are robust for different qPCR sensitivity levels assumed. For *Ascaris*, the sensitivity of qPCR has no effect for qPCR sensitivity levels greater than 75% when the prevalence threshold to declare interruption of transmission is less than or equal to 7.5%. Whilst for hookworm, PPV values remain over 0.85 if the sensitivity of qPCR is 75% for hookworm with a 1–2% prevalence threshold to declare elimination. The hookworm expulsion data was only performed during the follow-up study and was therefore not very successful (only 11 individuals with hookworm were detected) [16]. Expulsion data from areas which are close to the interruption of transmission are not currently available, but some studies have recently collected, or are in the process of collecting, such information (i.e TUMIKIA and DeWorm3). There is a clear need to acquire more data regarding the sensitivity of diagnostic tools and qPCR particularly in low-intensity settings. Animal models may be a good alternative as worm counts are more reliable post-mortem [50].

One of the main disadvantages of qPCR is the costs involved. However, there has been continued progression in the development of molecular techniques, resulting in lower economic costs per test [51]. The per test direct costs should not be considered in isolation, since indirect costs such as labour should also be considered [52]. In addition, the sensitivity of KK, particularly for hookworm, also depends on the time between stool collection and processing of the samples, as hookworm eggs/larvae degrade quickly over time [53]. Moreover, qPCR results show less between-sample variation than KK, making it possible to use just one sample, thus perhaps making qPCR more cost-effective [17].

The likelihood of achieving interruption and the ability to detect this depends on many factors, such as the baseline prevalence (i.e. the intrinsic transmission intensity in a given location). In the simulations described in this paper, the transmission parameter (R_0_), severity of density dependence in fecundity, age-related changes in exposure to infection and the value of the aggregation parameter *k* were fixed. Clustered randomized trials (such as DeWorm3 and TUMIKIA) are often based on clusters of villages. These villages are likely to exhibit variability in the intrinsic or pristine transmission intensity and in STH species mix. By grouping these villages into clusters, it becomes more difficult not only to achieve elimination, but also to measure its occurrence village by village. The PPV value drops substantially as the number of villages per cluster increases [9] and a lower prevalence threshold may be needed to increase the PPV and NPV values and hence improve the certainty in declaring elimination. Therefore, the optimal threshold may vary in different spatial and country settings.

One specific weakness in our simulation studies is the assumption that the aggregation parameter *k* is independent of the prevailing prevalence of infection. If the value of *k* reduces (worms become more aggregated as prevalence falls) perhaps due to persistent non-compliers to treatment, different threshold prevalence may be appropriate in defining an elimination threshold. This aspect is the subject of further study.

Countries which move from morbidity control programmes towards transmission elimination should carefully consider the difference in prevalence estimates due to increased sampling and the higher sensitivity gained from the new diagnostic tools. In determining the impact of MDA when moving towards elimination goals, it is important to sample community-wide and not just in SAC. For example, for hookworm, intensity and prevalence are normally found to be higher amongst adults [32]. However, *Ascaris* infections may be lower when measured community-wide as the highest infections are typically found in SAC. In such measures, due note must be taken of community demography. Due to the intensified sampling and more sensitive diagnostics, the measured prevalence will be higher than currently measured during LF transmission assessment surveys (TAS), which are commonly based on one slide of KK and only recorded in SAC. The higher-than-expected prevalence may alarm programme managers involved in elimination trials and result in a higher coverage or frequency of MDA than specified in the programme design specifications, which consequently could harm the experimental design. Good communication will be necessary to tackle these issues.

Temperature and humidity affect the survival of the free-living stages in the environment and this can be important for the timing of MDA. However, the impact may differ between settings and the data needed to quantify the impact are very limited at present [54].

The number of eggs released per female worm differs between the helminth species. *Ascaris* female worms are thought to release approximately 200,000 eggs per day [55], whilst female hookworms are thought to produce approximately 30,000 eggs per day [56]. Given the large number of eggs produced, we do not expect worm debris to contribute significantly to the DNA detected by PCR techniques. Hence, any detected DNA should mainly derive from eggs produced by the female worms. However, the sensitivity of qPCR in cases where no eggs are produced is currently unknown. qPCR techniques may produce positive results when worms in a host do not produce eggs. For example, this may be the case for *Ascaris* when a host has only male worms, or for hookworm when both sexes do not reside within a host. Recognising that such events may occur is much easier than presenting solutions to negate such measurement errors.

Serological assays may be an alternative to qPCR. Serology assays, measuring specific antibody responses to defined worm antigens, can overcome the problem of identifying immature parasitic infections [57,58]. These methods, including enzyme-linked immunosorbent assay (ELISA), can provide quantitative measurements of worm infection, through specific antibody titres, of both mature and immature worms in the intestine, as well as circulating larvae. At present, however, little is understood about whether such antibody titres reflect past or current infection, or both. They obviously can detect circulating antibodies produced by previous, as well as current infections. In the absence of reinfection these antibodies are generally thought to have a short half-life of up to six months but much uncertainty surrounds such figures as does the role of memory cells in stimulating new antibody production [59,60]. Serology assays may become very valuable tools in the future when testing for elimination of infection in a community, one or more years after the end of an MDA programme. At present, however, too much uncertainty surrounds the question of what antibody titres precisely measure and the variability in response patient-by-patient to a defined antigenic exposure.

In conclusion, diagnostic tests with improved sensitivity are increasingly important as we move towards a goal of transmission interruption for the helminth NTDs. However, there are currently no “gold standards” for the detection of STH infections aside from the now very old faecal egg detection methods by microscopy or floatation [16,30,42,51]. The use of qPCR as a diagnostic tool for STH is a very recent development. At present there is a need for standards plus protocols to be agreed on internationally (e.g. by WHO) and clearly defined for the DNA to be detected plus sample preparation and replication. More research is needed to investigate whether qPCR can detect juvenile infections. If this is the case, and it is thought to be unlikely, the elimination threshold should be higher than specified. However, even though the use of qPCR presents a number of important challenges, its high sensitivity makes it a very valuable tool in working towards the elimination of STH species.

## Supporting information

S1 Fig Comparison of stochastic individual-based model runs (grey lines) and the deterministic model (red line). Prevalence is the measured prevalence as described by Coffeng et al., (2017) and Truscott et al., (2017) [9,35].
(TIF)

S2 Fig Bayesian fitting to mean worm burden (mu) and aggregation parameter k for *Ascaris*. (TIF)

S3 Fig Sample size calculations depending on test sensitivity. (TIF)

S1 R Code Fiiting a negative binomial distribution to worm count data (with MCMC) to estimate the aggregation parameter k. (DOCX)

S1 Table Model parameters of the simulation model for Ascaris and hookworm. Please note, the major differences between the diseases are reflected by the female worm fecundity and reservoir decay rate.
(DOCX)
