# Supplementary figures and images for: Testing for soil-transmitted helminth transmission elimination: Analysing the impact of the sensitivity of different diagnostic tools
Source: PLoS Negl Trop Dis. 2018 Jan 18;12(1):e0006114. doi: 10.1371/journal.pntd.0006114 (PMC5773090; doi:10.1371/journal.pntd.0006114)

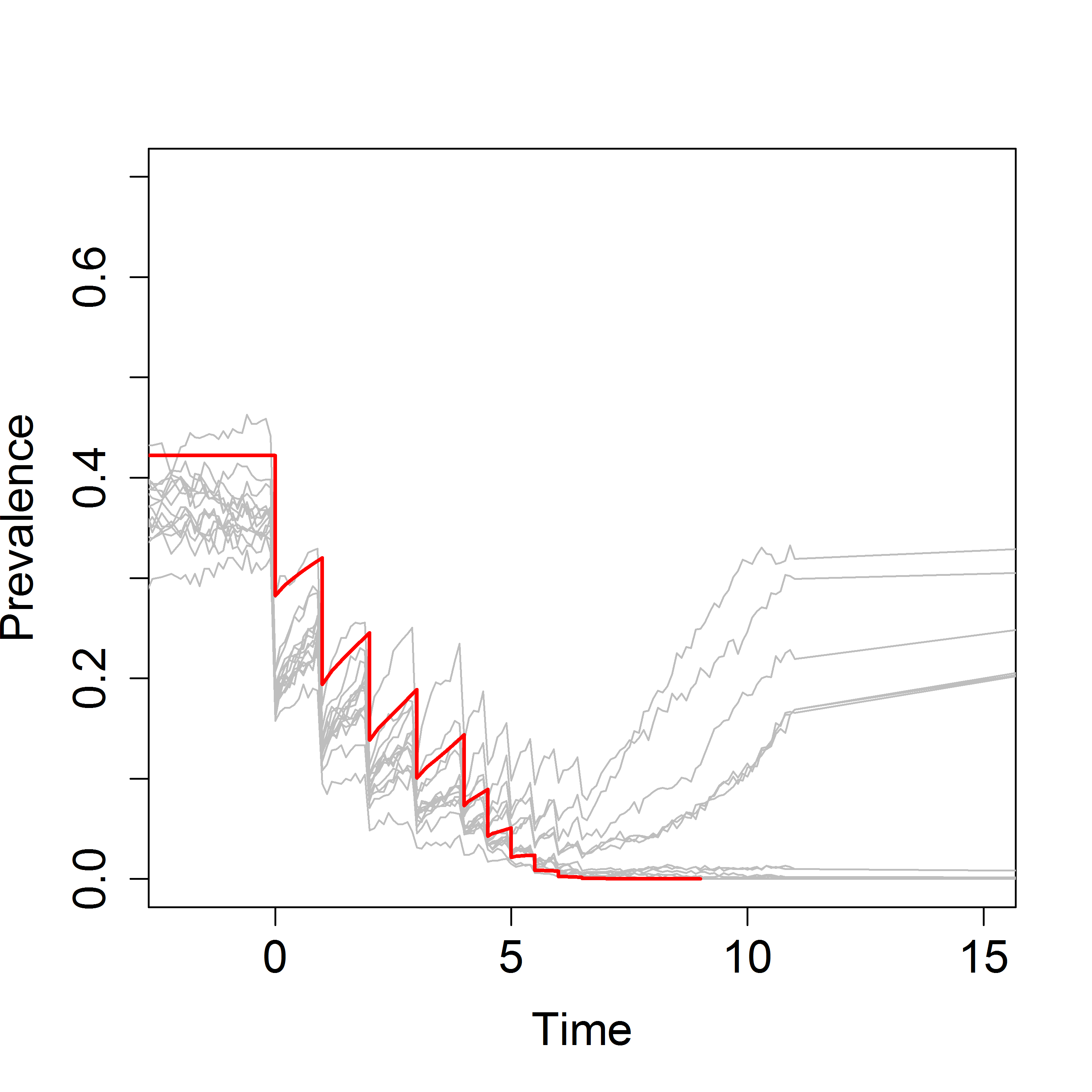

Supplement: S1 Fig — Prevalence is the measured prevalence as described by Coffeng et al., (2017) and Truscott et al., (2017) [9,35]. (TIF) [file pntd.0006114.s001.tif]

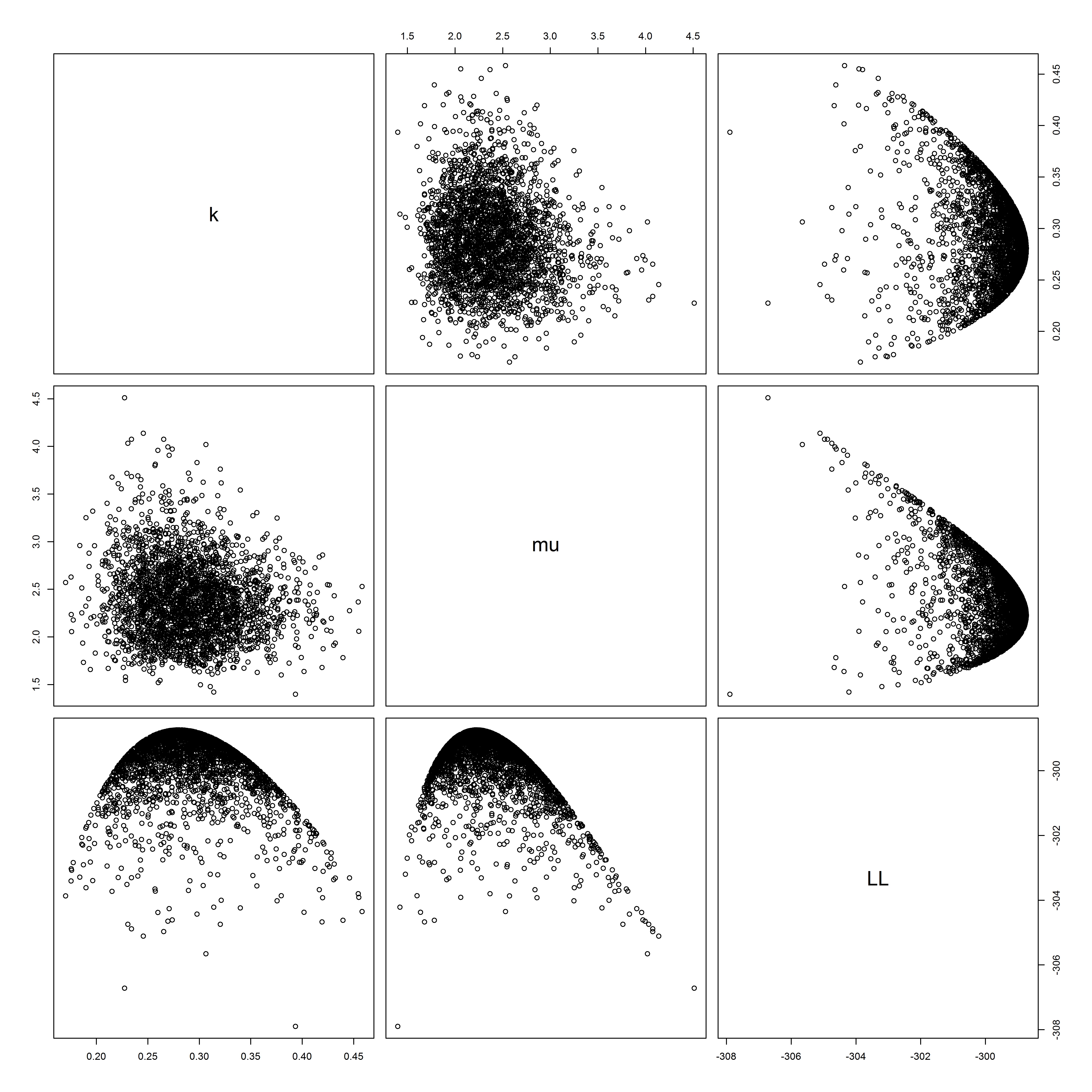

Supplement: S2 Fig — (TIF) [file pntd.0006114.s002.tif]

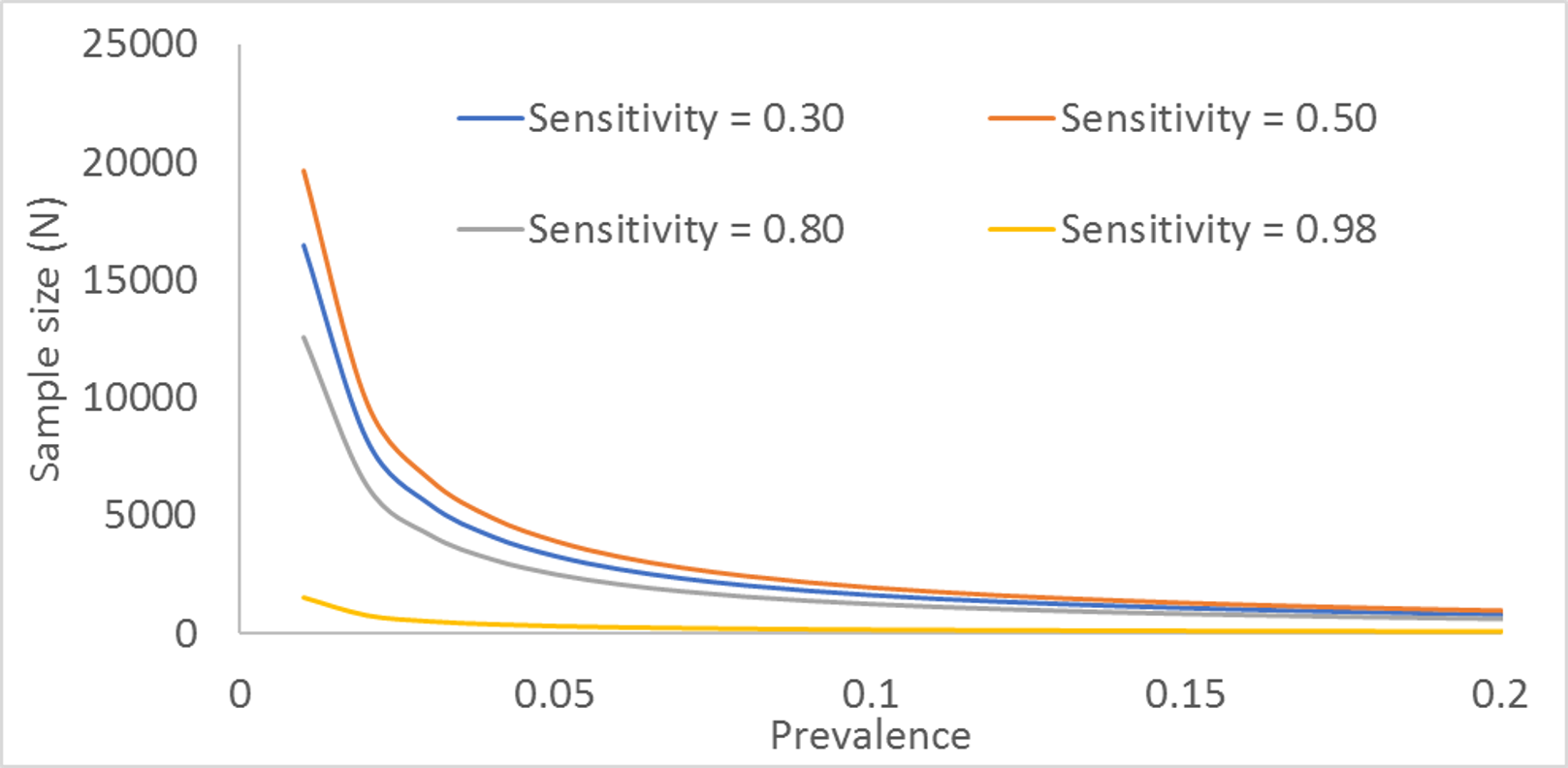

Supplement: S3 Fig — (TIF) [file pntd.0006114.s003.tif]
