# Supplementary material for: Testing for soil-transmitted helminth transmission elimination: Analysing the impact of the sensitivity of different diagnostic tools
Source: PLoS Negl Trop Dis. 2018 Jan 18;12(1):e0006114. doi: 10.1371/journal.pntd.0006114 (PMC5773090; doi:10.1371/journal.pntd.0006114)
Supplement: S1 R Code — (DOCX) [file pntd.0006114.s004.docx]

**Supplementary material: R code to fit a negative binomial distribution to worm count data (with MCMC) to estimate the aggregation parameter *k*.**

require("mcmc")

worms<-data$BL_worms

mean(worms)

getLL <- function(param)

{

k<-param[1]

mu<-param[2]

if(mu<0.01) return(-Inf)

LLiTot<-sum(dnbinom(worms,mu=mu,size=k,log=T),na.rm=T)

}

# Set initial parameters for lambda and k, store in vector param

k<-0.3

mu<-3

param<-c(k,mu)

out <- metrop(getLL,initial=param,nbatch=5000,nspac=1,scale=c(0.025,0.5))

LL <- apply(out$batch,MARGIN=1,getLL)

MCMC_LL_Worms <- cbind(out$batch,LL)

colnames(MCMC_LL_Worms)<-c("k","mu","LL")

pairs(MCMC_LL_Worms)
