# Supplementary material for: Testing for soil-transmitted helminth transmission elimination: Analysing the impact of the sensitivity of different diagnostic tools
Source: PLoS Negl Trop Dis. 2018 Jan 18;12(1):e0006114. doi: 10.1371/journal.pntd.0006114 (PMC5773090; doi:10.1371/journal.pntd.0006114)
Supplement: S1 Table — Please note, the major differences between the diseases are reflected by the female worm fecundity and reservoir decay rate. (DOCX) [file pntd.0006114.s005.docx]

**Table S1. Model parameters of the simulation model for Ascaris and hookworm. Please note, the major differences between the diseases are reflected by the female worm fecundity and reservoir decay rate.**

| **Model parameter description** | ***Ascaris*** | **Hookworm** |
| --- | --- | --- |
| Transmission rate | R_0_ = 2.12 | R_0_ = 2.2 |
| Aggregation of parasites in hosts | *$k$* = 0.285 (fig S2) | *$k$*  = 0.35 [35] |
| Relative exposure and contribution to the reservoir (assuming no difference between males and females) | - 0-2 years old: 0.22 - 2-5 years old: 1.88 - 5-15 years old: 1 - 15+ years old: 0.53 | - 0-15 years old: 0.12 - 15-25 years old: 1 - 25+ years: 0.067 [35] |
| Average worm lifespan | 1 (assuming an exponential distribution) | 2 years (assuming an exponential distribution) [35] |
| Female worm fecundity | $\gamma$ = 0.07 | $\gamma$ = 0.02 (assuming exponential saturation) [35] |
| Reservoir decay rate | mean = 2 months | mean = 12 days [49] |
| Drug efficacy | 0.950 | 0.940 [35] |
